# Lipoprotein(a) level predicts the development of nonalcoholic fatty liver disease in Korean adults: A retrospective longitudinal study

**DOI:** 10.1097/MD.0000000000038340

**Published:** 2024-05-31

**Authors:** Ji Sun Nam, Kahui Park, Su Jung Baik, Jong Suk Park

**Affiliations:** a Division of Endocrinology and Metabolism, Department of Internal Medicine, Yonsei University College of Medicine, Seoul, Republic of Korea; b Severance Institute for Vascular and Metabolic Research, Yonsei University College of Medicine, Seoul, Republic of Korea; c Healthcare Research Team of Health Promotion Center, Gangnam Severance Hospital, Yonsei University College of Medicine, Seoul, Republic of Korea.

**Keywords:** hepatic fibrosis, lipoprotein(a), nonalcoholic fatty liver disease

## Abstract

Nonalcoholic fatty liver disease (NAFLD) is a highly prevalent condition in the general population. Although recent studies have demonstrated a link between NAFLD and lipoprotein(a), a low-density lipoprotein-like particle synthesized in the liver, its precise physiological role and mechanism of action remain unclear. This study aimed to investigate the relationship between lipoprotein(a) levels and development of NAFLD and hepatic fibrosis in Korean adults. A total of 1501 subjects who underwent abdominal ultrasonography at least twice as part of a health checkup program were enrolled. Biochemical and ultrasonography results were analyzed longitudinally, and the degree of hepatic fibrosis was calculated in subjects with NAFLD using serum biomarkers, such as fibrosis-4 (FIB-4). During the 3.36-year follow-up period, 352 patients (23.5%) were diagnosed with NAFLD. The subjects were categorized into 4 groups based on their lipoprotein(a) levels. Remarkably, the incidence of NAFLD decreased as the lipoprotein(a) levels increased. Following logistic regression analysis and adjustment for various risk factors, the odds ratio for the development of NAFLD was 0.625 (95% CI 0.440–0.888; *P* = .032) when comparing the highest to the lowest tertile of lipoprotein(a). However, no significant association was observed between the occurrence of hepatic fibrosis and lipoprotein(a) levels in subjects with NAFLD. Lipoprotein(a) levels have been identified as a significant predictor of NAFLD development. Additional large-scale studies with extended follow-up periods are required to better understand the effect of lipoprotein(a) on NAFLD and hepatic fibrosis.

## 1. Introduction

Fatty liver disease has emerged as a significant global health issue, mostly owing to lifestyle changes. Among the various types of fatty liver disease, nonalcoholic fatty liver disease (NAFLD) is particularly concerning, as it can develop irrespective of alcohol abuse, encompassing a range of conditions from simple steatosis to steatohepatitis and potentially progressing to advanced fibrosis and cirrhosis.^[1,2]^ There is copious evidence that establishes a close relationship between NAFLD and not only metabolic diseases, such as obesity and insulin resistance, but also cardiovascular disease.^[3–6]^

Lipoprotein(a) is a complex composed of a low-density lipoprotein (LDL)-like particle containing apolipoprotein B-100 linked by a disulfide bond to apolipoprotein(a).^[7,8]^ It is an atherogenic lipoprotein that is synthesized by the liver and is inversely correlated with triglyceride levels.^[9]^ Recent research has considered lipoprotein(a) a promising new biomarker in the fields of lipidology, preventive cardiology, and fatty liver disease.^[10,11]^

Previous research has shown a relationship between lipoproteins (a) and fatty liver disease. Although substantial evidence suggests an inverse relationship between the 2, some studies have reported inconclusive results.^[9,12–14]^ To date, a systematic review of 10 observational studies reported conflicting and inconclusive associations between lipoprotein(a) levels and hepatic steatosis.^[15]^ Furthermore, while liver fibrosis is the most significant predictor of NAFLD outcomes and liver-related mortality, the importance of early identification of fibrosis has been emphasized.^[16–19]^ However, only 2 cross-sectional studies have investigated the relationship between lipoprotein(a) levels and hepatic fibrosis.^[20,21]^ No previous studies have examined the relationship between lipoprotein(a) levels and the progression of NAFLD and hepatic fibrosis. Therefore, the current study aimed to examine the association between baseline lipoprotein(a) levels and the future development of NAFLD and hepatic fibrosis in Korean adults.

## 2. Methods

### 2.1. Study population

This retrospective longitudinal study examined 12,599 individuals who underwent abdominal ultrasonography as part of a self-referred checkup program at the Gangnam Severance Hospital Health Promotion Center in Seoul, Korea. A total of 3293 participants who underwent at least 2 abdominal ultrasonography procedures were enrolled in the study. Subjects diagnosed with diabetes mellitus, fatty liver disease, or other liver diseases, including hepatitis, liver cirrhosis, and hepatocellular carcinoma, at baseline (n = 1343) were excluded from the analysis. Additionally, participants with missing data (n = 187) and those taking medications for dyslipidemia (n = 158) were excluded, as were individuals with daily alcohol consumption exceeding 30 g for men and 20 g for women (n = 104). Ultimately, 1501 participants were included in the analysis. This study was approved by the Institutional Review Board of Yonsei University College of Medicine. The requirement for informed consent was waived owing to the retrospective nature of the study, and the data were stored anonymously.

### 2.2. Anthropometric measurements and laboratory assessment

Height and weight were measured to calculate body mass index (BMI, kg/m^2^). Experienced technicians measured the systolic blood pressure (SBP) and diastolic blood pressure (DBP) after a 5-minute rest using an automated blood pressure monitor (HEM-7080IC; Omron Healthcare, Lake Forest, IL), with the patient arm placed at the same level as the heart.

After an 8-hours fasting period, blood samples were collected from all the subjects. The samples were then immediately centrifuged, and the serum was stored at −70°C until further analysis. Fasting plasma glucose (FPG), total cholesterol (TC), high-density lipoprotein cholesterol (HDL-C), triglyceride (TG), aspartate aminotransferase (AST), alanine aminotransferase (ALT), and gamma-glutamyl transpeptidase (GGT) levels were assessed by enzymatic procedures using an automated chemistry analyzer (Hitachi 7600-120, Tokyo, Japan). Low-density lipoprotein cholesterol (LDL-C) levels were calculated using the Friedewald formula. The levels of the hepatitis B surface antigen and anti-hepatitis C virus antibodies were measured using a Roche E-170 device (Roche Diagnostics). Serum lipoprotein(a) levels were quantitatively measured by rate nephelometry (IMMAGE System; Beckman Coulter). The inter-assay CV% for lipoprotein(a) was 9.03% and 2.75% at low and high concentrations, respectively, whereas the intra-assay CV% was 1.77% and 0.82% at low and high concentrations, respectively.

The social and medical history of each participant was obtained using a self-administered questionnaire that included questions regarding smoking, alcohol status, medications, and history of other diseases. Diabetes mellitus was diagnosed on the basis of a history of diabetes or the diagnostic standards of the American Diabetes Association. Subjects with SBP and/or DBP ≥ 140/90 mm Hg or those currently taking antihypertensive medications were classified as hypertensive. Current smokers were defined as those who had smoked cigarettes regularly for at least 6 months. Regular exercise was characterized by moderate-intensity physical activity for more than 30 minutes at least 3 times per week.

### 2.3. Assessment of fatty liver disease and hepatic fibrosis

Fatty liver disease was diagnosed based on abdominal ultrasonography performed using a 3.5-MHz transducer (HDI 5000, Philips, Bothell, WA). One of the 3 experienced radiologists, who was blinded to the subjects’ clinical information, performed abdominal ultrasonography. The present study considered any degree of fat accumulation in the liver to be NAFLD.

Among subjects with NAFLD, the degree of hepatic fibrosis was calculated using serum biomarkers and the following formula: fibrosis-4 (FIB-4) = age (years) × AST [U/L]/(platelets [10^9^/L] × (ALT [U/L])^1/2^). Significant hepatic fibrosis was defined as FIB-4 advanced > 1.45.^[22–24]^

### 2.4. Statistical analysis

Continuous variables are presented as the mean ± standard deviation. Intergroup comparisons were performed using Student *t* test or one-way ANOVA. Categorical variables with percentages were compared using the chi-squared test. The association between NAFLD progression and lipoprotein(a) levels was assessed using a logistic regression analysis. Covariates in the multivariable model were selected based on clinical importance, with statistical significance set at *P* ≤ .20 or smaller for univariate analysis. After adjusting for confounding variables, multivariate logistic regression analysis was used to estimate the odds ratios (OR) and associated 95% confidence intervals (CI) for NAFLD based on the lipoprotein(a) levels. All statistical analyses in the present study were conducted using SPSS for Windows 25.0 (SPSS Inc., Chicago, IL), with *P* values <.05 indicating statistical significance.

## 3. Results

### 3.1. Baseline characteristics

The mean age of the 1501 subjects at baseline was 53.1 ± 9 years, with 95.1% of the subjects being men (n = 1428). The subjects were then stratified into 4 groups according to lipoprotein(a) levels. The clinical characteristics and biochemical parameters of the study participants are summarized in Table 1. The mean observation time was 3.36 years. Accordingly, subjects with higher lipoprotein(a) levels were older and had higher TC and LDL-C levels, but lower FPG, TG, and γGT levels than those with lower lipoprotein(a) levels. No difference in the prevalence of hypertension was found according to lipoprotein(a) levels (Table 1).

**Table 1:** Baseline characteristics of study subjects according to lipoprotein(a).

	Q1	Q2	Q3	Q4	*P* value
N	382	373	371	375	
Age (yr)	52.0 ± 9.6	52.5 ± 9.5	53.3 ± 9.2	54.4 ± 9.9	.004
Sex (M/F)	365/17	359/14	351/20	353/22	.537
BMI (kg/m^2^)	23.6 ± 2.7	23.5 ± 2.3	23.5 ± 2.5	23.4 ± 2.7	.753
SBP (mm Hg)	124.5 ± 13.6	122.7 ± 12.8	123.8 ± 14.3	124.0 ± 13.5	.328
DBP (mm Hg)	78.7 ± 9.4	77.1 ± 8.5	78.5 ± 9.7	77.9 ± 9.5	.070
FPG (mmol/L)	5.54 ± 1.11	5.46 ± 0.80	5.41 ± 0.85	5.36 ± 0.65	.047
TC (mmol/L)	4.92 ± 0.87	4.98 ± 0.85	5.10 ± 0.89	5.20 ± 1.03	<.001
TG (mmol/L)	1.53 ± 1.00	1.26 ± 0.61	1.23 ± 0.55	1.21 ± 0.55	<.001
HDL-C (mmol/L)	1.31 ± 0.32	1.32 ± 0.30	1.33 ± 0.30	1.34 ± 0.33	.390
LDL-C (mmol/L)	3.04 ± 0.81	3.10 ± 0.84	3.19 ± 0.78	3.29 ± 0.89	<.001
AST (U/L)	25.3 ± 10.4	24.7 ± 9.0	24.3 ± 8.4	24.9 ± 12.9	.586
ALT (U/L)	25.7 ± 13.6	25.4 ± 15.9	23.5 ± 13.2	25.4 ± 34.4	.487
GGT (U/L)	40.4 ± 42.0	35.0 ± 34.7	33.7 ± 28.2	33.2 ± 26.0	.010
Lp(a) (mg/L)	0.47 ± 0.17	1.03 ± 0.19	1.78 ± 0.28	4.80 ± 2.69	<.001
Hypertension (%)	80 (20.9)	77 (20.6)	77 (20.8)	73 (19.5)	.927
Smoking (%)	91 (23.8)	85 (22.8)	89 (24.0)	87 (23.2)	.952
Exercise (%)	166 (43.5)	166 (44.5)	186 (50.1)	175 (46.7)	.213
Observation time (years)	3.33 ± 1.68	3.29 ± 1.65	3.45 ± 1.62	3.38 ± 1.66	.600

Data are presented as mean ± standard deviation or number (percentage). ALT = alanine aminotransferase, AST = aspartate aminotransferase, BMI = body mass index, DBP = diastolic blood pressure, Exercise = regular moderate-intensity exercise, FPG = fasting plasma glucose, GGT = gamma-glutamyl transpeptidase, HDL-C = high-density lipoprotein cholesterol, LDL-C = low-density lipoprotein cholesterol, Lp(a) = lipoprotein(a), SBP = systolic blood pressure, Smoking = current smoker, TC = total cholesterol, TG = triglyceride.

### 3.2. Comparison of variables in relation to NAFLD development

The subjects were divided into 2 groups based on the development of NAFLD, as shown in Table 2. It was observed that subjects who developed NAFLD were younger in age and had higher baseline BMI, SBP, DBP, FPG, TG, AST, ALT, and GGT levels than those who did not develop NAFLD. Additionally, the NAFLD group had lower lipoprotein(a) levels.

**Table 2:** Comparison of baseline characteristics according to development of NAFLD.

	All	No NAFLD	NAFLD	*P* value
N	1501	1149	352	
Age (yr)	53.1 ± 9.6	53.4 ± 9.8	52.2 ± 9.0	.045
Sex (M/F)	1428/73	1088/61	340/12	.159
BMI (kg/m^2^)	23.5 ± 2.6	23.3 ± 2.5	24.3 ± 2.6	<.001
SBP (mm Hg)	123.7 ± 13.6	123.3 ± 13.7	125.2 ± 13.3	.020
DBP (mm Hg)	78.1 ± 9.3	77.7 ± 9.2	79.2 ± 9.4	.002
FPG (mmol/L)	4.95 ± 0.87	5.41 ± 0.84	5.58 ± 0.98	.005
TC (mmol/L)	5.05 ± 0.92	5.04 ± 0.90	5.08 ± 0.98	.488
TG (mmol/L)	1.31 ± 0.71	1.24 ± 0.66	1.53 ± 0.79	<.001
HDL-C (mmol/L)	1.33 ± 0.31	1.35 ± 0.31	1.25 ± 0.32	<.001
LDL-C (mmol/L)	3.16 ± 0.84	3.15 ± 0.82	3.17 ± 0.89	.655
AST (U/L)	24.8 ± 10.3	24.3 ± 9.0	26.3 ± 13.8	.002
ALT (U/L)	25.0 ± 21.2	23.3 ± 12.3	30.7 ± 37.2	<.001
GGT (U/L)	35.6 ± 33.5	32.8 ± 30.4	44.8 ± 40.7	<.001
Lp(a) (mg/L)	2.01 ± 2.16	2.15 ± 2.34	1.56 ± 1.31	<.001
Hypertension (%)	307 (20.5)	219 (19.1)	88 (25.0)	.019
Smoking (%)	352 (23.5)	264 (23.0)	88 (25.0)	.340
Exercise (%)	693 (46.2)	536 (46.6)	157 (44.6)	.440
Observation time (yr)	3.36 ± 1.66	3.33 ± 1.69	3.47 ± 1.52	.181

Data are presented as mean ± standard deviation or number (percentage). ALT = alanine aminotransferase, AST = aspartate aminotransferase, BMI = body mass index, DBP = diastolic blood pressure, Exercise = regular moderate-intensity exercise, FPG = fasting plasma glucose, GGT = gamma-glutamyl transpeptidase, HDL-C = high-density lipoprotein cholesterol, LDL-C = low-density lipoprotein cholesterol, Lp(a) = lipoprotein(a), NAFLD = nonalcoholic fatty liver disease, SBP = systolic blood pressure, Smoking = current smoker, TC = total cholesterol, TG = triglyceride.

### 3.3. NAFLD development according to lipoprotein(a) quartiles

**Figure 1.**
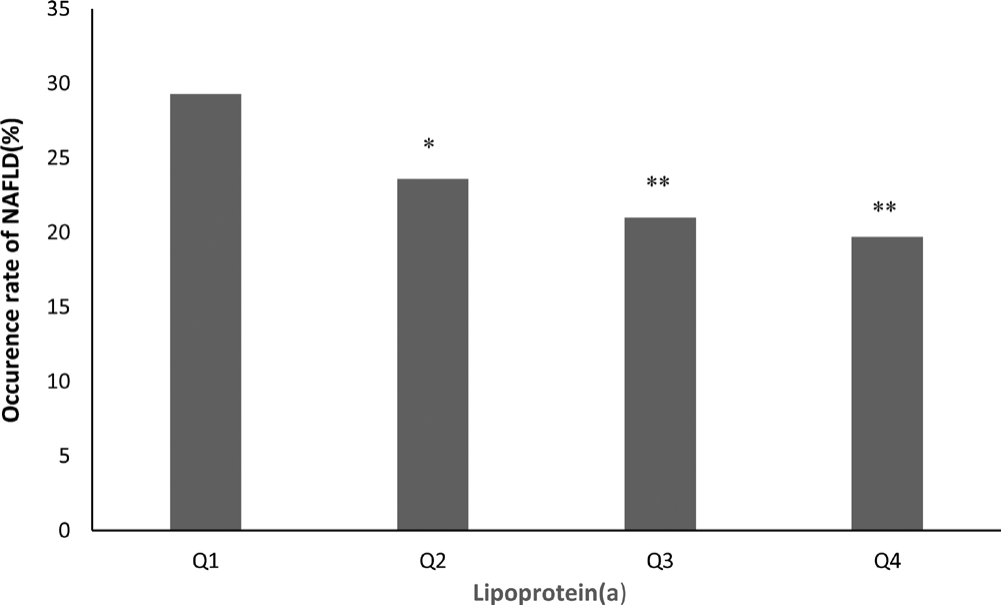
NAFLD development according to lipoprotein(a) quartiles. The occurrence rate of NAFLD decreased across the quartiles of lipoprotein(a) (Q1: lowest Lp(a) quartile, Q4: highest Lp(a) quartile). Statistical analysis was performed using the chi-squared test, comparing each group to Q1 (*P < .05, **P < .01).

Figure 1 shows that NAFLD development decreased across quartiles of lipoprotein(a) at baseline. Subjects with higher lipoprotein(a) levels had lower rates of NAFLD.

### 3.4. Association between NAFLD development and lipoprotein(a)

Univariate logistic regression analysis revealed that age, BMI, SBP, DBP, FPG, TG, HDL-C, hypertension, and lipoprotein(a) levels were significantly associated with the development of NAFLD (Table 3). Using Q1 as a reference, multivariate logistic regression analysis revealed that lipoprotein(a) levels for Q2, Q3, and Q4 decreased the OR for the development of NAFLD. This relationship remained significant, even after adjusting for sex, age, and other risk factors (Table 4).

**Table 3:** Association between the development of NAFLD as a dependent variable and other metabolic parameters.

	OR (95% CI)	*P* value
Age	0.987 (0.975–1.000)	.045
Sex (male)	1.589 (0.845–2.985)	.150
BMI	1.163 (1.109–1.220)	<.001
SBP	1.010 (1.002–1.019)	.021
DBP	1.021 (1.008–1.034)	.002
FPG	1.010 (1.003–1.017)	.007
TC	1.001 (0.998–1.005)	.488
TG	1.006 (1.004–1.007)	<.001
HDL-C	0.971 (0.960–0.982)	<.001
LDL-C	1.001 (0.997–1.005)	.655
Hypertension	1.411 (1.064–1.872)	.017
Smoking	1.155 (0.871–1.532)	.318
Exercise	0.905 (0.703–1.165)	.437
Observation time	1.050 (0.977–1.129)	.181
Lp(a)		.010
Q2 vs Q1	0.744 (0.538–1.030)	
Q3 vs Q1	0.642 (0.460–0.895)	
Q4 vs Q1	0.593 (0.423–0.830)	

BMI = body mass index, CI = confidence interval, DBP = diastolic blood pressure, Exercise = regular moderate-intensity exercise, FPG = fasting plasma glucose, HDL-C = high-density lipoprotein cholesterol, LDL-C = low-density lipoprotein cholesterol, Lp(a) = lipoprotein(a), OR = odds ratio, SBP = systolic blood pressure, Smoking = current smoker, TC = total cholesterol, TG = triglyceride.

**Table 4:** Development of NAFLD odds ratios and 95% confidence intervals based on the lipoprotein(a) quartiles.

	OR (95% CI)				*P* for trend
	Q1	Q2	Q3	Q4	
Unadjusted	1	0.744	0.642	0.593	.010
		(0.538–1.030)	(0.460–0.895)	(0.423–0.830)	
Age & sex adjusted	1.00	0.746	0.653	0.622	.017
		(0.538–1.033)	(0.467–0.911)	(0.436–0.857)	
Multivariable adjusted*	1.00	0.780	0.656	0.625	.032
		(0.558–1.091)	(0.465–0.925)	(0.440–0.888)	

OR, odds ratio.

* Age, sex, BMI, SBP, DBP, FPG, TG, HDL-C, presence of hypertension, and observation time.

### 3.5. Association between hepatic fibrosis and lipoprotein(a)

Among the subjects with NAFLD, the incidence of hepatic fibrosis, defined as FIB-4 > 1.45, was not significantly associated with lipoprotein(a) quartiles (29.5%, 30.7%, 44.9%, and 36.5%; *P = *.134). After dividing participants into 3 groups according to the occurrence of NAFLD and hepatic fibrosis, no significant differences in baseline lipoprotein(a) values were observed among the following 3 groups: no NAFLD, NAFLD without fibrosis, and NAFLD with fibrosis (19.71 ± 21.90, 19.86 ± 19.20, 16.84 ± 3.19, *P* = .302).

## 4. Discussion

The current study demonstrated an association between lipoprotein(a) levels and future development of NAFLD in Korean adults. Even after adjusting for other risk factors, lipoprotein(a) levels remained independently associated with the development of NAFLD. To the best of our knowledge, only a few studies have longitudinally investigated the occurrence of NAFLD, and the current study is the first to analyze the development of NAFLD along with hepatic fibrosis markers.

As expected, the current study found that lipoprotein(a) levels were significantly correlated with metabolic parameters, a finding consistent with those of previous studies.^[12,14]^ Lipoprotein(a) also showed a significant association with new-onset NAFLD; however, it did not predict the development of hepatic fibrosis (defined as FIB-4 > 1.45).

To date, several studies have reported an association between lipoprotein(a) levels and NAFLD development, but their results remain controversial. Yang et al reported that high lipoprotein(a) levels are an independent protective factor against NAFLD.^[9]^ Similarly, an inverse association between lipoprotein(a) and NAFLD was observed in another study, but it failed to reveal whether lipoprotein(a) was an independent predictive marker.^[12]^ In contrast, Zhang et al reported a positive correlation between lipoprotein(a) levels and NAFLD severity.^[13]^ All 3 previous studies had limitations, owing to their cross-sectional nature. In a retrospective longitudinal study performed on Korean subjects participating in a health checkup program, Jung et al reported that lipoprotein(a) levels were inversely associated with the presence of fatty liver disease^[14]^; however, the study did not exclude drinkers, and alcoholic fatty liver disease was included in the analysis.

NAFLD is considered a significant condition owing to its potential to progress to cirrhosis, hepatocellular carcinoma, and the need for liver transplantation. Nevertheless, not all patients with NAFLD experience such a progression, which emphasizes the importance of hepatic fibrosis in the screening and intensive management of high-risk patients. Few studies have investigated the relationship between the lipoprotein(a) levels and hepatic fibrosis. According to Konoshi et al, lipoprotein(a) levels in advanced fibrosis were lower than those in non-advanced fibrosis.^[20]^ Additionally, Meroni et al reported that low lipoprotein(a) levels predict hepatic fibrosis in patients with NAFLD.^[21]^ These cross-sectional studies have demonstrated the negative relationship between the 2. Although the mechanism underlying the relationship between lipoprotein(a) levels and hepatic fibrosis has not been fully elucidated, Meroni et al explained that lipoprotein(a) subunits are synthesized in the liver and that the concentration of lipoprotein(a) is primarily regulated by hepatic apolipoprotein(a) synthesis; thus, the decrease in liver function caused by hepatic fibrosis can affect serum lipoprotein(a) levels. An alternative explanation for the inverse correlation between the expression of lipoprotein(a) and fibrogenic genes could be attributed to the diminished inhibition of transforming growth factor- β (TGF-β) by lipoprotein(a). The loss of the inhibitory effect on TGF-β due to low lipoprotein(a) levels results in enhanced smooth muscle cell activation and migration.^[21,25]^ Liver biopsy remains the most reliable method for diagnosing hepatic fibrosis. however, it is invasive and impractical as a screening tool. In recent years, alternative noninvasive scoring systems, such as FIB-4, have become popular for evaluating hepatic fibrosis.^[2,26,27]^ This study examined the association between baseline lipoprotein(a) levels and the occurrence of hepatic fibrosis using FIB-4, revealing that lipoprotein(a) levels are not effective in predicting the development of hepatic fibrosis in individuals with NAFLD. This may be attributed to several factors that contribute to the progression of NAFLD to hepatic fibrosis, including genetic variants of lipoprotein(a), and the time required for this progression.^[17,28,29]^

Our study has several limitations. First, the results may not be generalizable, given that most subjects were men, and the study was conducted at a single center. Second, liver biopsy, which is essential for assessing NAFLD and hepatic fibrosis, was not performed, which is a limitation of the FIB-4 index. Third, the study did not investigate the impact of insulin resistance because insulin levels were not assessed during routine health checkups. Finally, the mean follow-up period of this study was relatively short.

In conclusion, the current study found that lipoprotein(a) level is an independent predictor of NAFLD development. Further prospective larger-population studies including additional ethnic groups with longer follow-up periods and appropriate hepatic fibrosis assessment methods are warranted to verify the effects of lipoprotein(a) on hepatic fibrosis.

## Acknowledgments

We thank the Gangnam Severance Health Promotion Research team for supporting the construction of the registry of data from the Health Promotion Center of the Gangnam Severance Hospital. We thank the Biostatistics Collaboration Unit, Department of Research Affairs, Yonsei University College of Medicine for database analysis and Enago writers for editing the English text.

## Author contributions

**Conceptualization:** Kahui Park, Jong Suk Park

**Data curation:** Ji Sun Nam, Kahui Park, Su Jung Baik, Jong Suk Park

**Investigation:** Ji Sun Nam, Kahui Park, Su Jung Baik, Jong Suk Park

**Methodology:** Ji Sun Nam, Kahui Park, Jong Suk Park

**Supervision:** Jong Suk Park

**Validation:** Ji Sun Nam, Su Jung Baik, Jong Suk Park

**Writing – original draft:** Ji Sun Nam, Kahui Park, Jong Suk Park

**Writing – review & editing:** Ji Sun Nam, Kahui Park, Jong Suk Park
